# Telomerase activator-65 and pomegranate peel improved the health status of the liver in aged rats; multi-targets involved

**DOI:** 10.22038/ijbms.2021.56670.12655

**Published:** 2021-06

**Authors:** Ameera Saeed Alshinnawy, Wael Mohamed El-Sayed, Ahmed AbdelAziz Sayed, Ahmed Mohamed Salem, AlShaimaa Mohamed Taha

**Affiliations:** 1 Department of Biochemistry, Faculty of Science, Ain Shams University, Cairo, Egypt. 11566; 2 Department of Zoology, Faculty of Science, Ain Shams University, Cairo, Egypt. 11566; 3 Children’s Cancer Hospital 57357, Cairo, Egypt

**Keywords:** Aging, Apoptosis, IGF1, Oxidative stress, Telomerase, Thioredoxin reductase

## Abstract

**Objective(s):**

This study was undertaken to investigate the efficacy of telomerase activator-65 (Ta-65) and pomegranate peel against aging-induced deteriorations in the liver.

**Materials and Methods:**

The rats were divided into four groups: control, aged, aged rats treated with Ta-65, and pomegranate orally for two months.

**Results:**

Aging significantly increased the serum levels of total protein, globulins, and protein carbonyl and reduced the insulin-like growth factor 1 (IGF-1). It also elevated the hepatic malondialdehyde and decreased the hepatic glutathione S-transferase (GST) activity. Aging elevated the expression of thioredoxin reductase1, telomerase reverse transcriptase, and cytochrome 3a1 in the liver; it increased the p53 protein level and elevated the activity of caspase-3 in the liver indicating the occurrence of apoptosis. The architecture of the liver deteriorated in the aged rats, as shown by both light and electron microscopy examinations. The liver of the aged rats had many apoptotic hepatocytes with shrunken nuclei. Many hepatocytes had dilated rough endoplasmic reticulum, many lysosomes, and many fat droplets. Administration of Ta-65 and pomegranate to the aged rats normalized most of the previous biochemical parameters and improved the liver architecture.

**Conclusion:**

Ta-65 and pomegranate have anti-aging activity through targeting multiple cellular pathways. It is also noteworthy that Ta-65 was superior to pomegranate in its alleviative effects.

## Introduction

Aging is a biological natural process causing homeostasis imbalance and deterioration in physiological functions, proliferation, and immune response control (1). During aging, excessive liver apoptosis has been identified, and in some cases liver fibrosis develops (1, 2). Aging is also characterized by dysfunction of many proteins such as insulin-like growth factor 1 (IGF-1), telomerase reverse transcriptase (TERT), and cytochrome P450 (3, 4).

Normally, IGF-1 helps the cells to adapt to the nutrient stress through regulating gene expression and protein modification. Unfortunately, the efficiency of IGF-1 declines by aging. Due to the deficiency in telomerase RNA component or TERT, telomere dysfunction has been observed in the livers of aged people and may result in chronic liver diseases or carcinoma (3). In aging, telomere dysfunction-induced hepatic apoptosis involving a p53-dependent pathway was previously reported (1). Telomere dysfunction mainly impacts the G1 phase *via* activating the DNA damage response checkpoints (5).

Natural ingredients with anti-oxidant, anti-inflammatory, and anti-apoptotic characteristics for preventing and/or treating aging have gained great attention. The genus *Astragalus* belongs to the *Fabaceae* legume family (6). The constituents of the dried roots of *Astragalus membranaceus* provide significant protection against injury in many organs (7). This is due to its polysaccharide and astragaloside bioactive constituents (8). *A. membranaceus *telomerase activator (TA-65) is an extract from *A. membranaceus *plant root. TA-65 has been identified as an effective telomerase activator (9, 10). Based on animal studies, no adverse effect was recorded after oral administration of Ta-65 at doses greater than 150 mg/kg in male and female rats, equivalent to 10,500 mg/day in a 70-kg individual, which is higher than the highest doses seen in human pharmacokinetic studies (11).

*Punica granatum* L. (pomegranate) fruits are widely consumed in many countries in the form of juice, wines, or crude fruit. Pomegranate fruit waste including peel has a substantial amount of phytonutrients with known potent health benefits including anti-oxidant and antimicrobial activities (12). Pomegranate peels represent 26%–30% of the fruit and have 92% of the total anti-oxidant activity of pomegranate (13) because of their large polyphenol content such as punicalagin (14), flavonoids (anthocyanins, catechins, and other complex flavonoids), and hydrolyzable tannins (punicalin, pedunculagin, punicalagin, and gallic and ellagic acid) (12, 15). Clinical trials in human subjects have reported the significant effect of pomegranate consumption in improving the defense against various forms of infections and inflammatory and non-inflammatory disorders. Pomegranate peel and its hydroalcoholic extracts have been considered safe for human health at orally administrated doses up to 2000 mg per day (12). 

Recently, both Ta-65 and *P. granatum *peel supplements were reported to alleviate male infertility and kidney dysfunction induced in rats by normal aging (16). These results encouraged us to study the efficacy of Ta-65 and pomegranate supplements against normal aging-induced hepatic apoptosis in rats *via* investigating the apoptotic pathway in liver tissue and reporting the liver histological alterations by light and electron microscopes.

## Materials and Methods

***Preparation of Ta-65 and pomegranate supplements***

The powders of Ta-65 supplement (RD Health Ingredients Co., China, CAS: 78574-94-4) and pomegranate (Wuhan HengHeDa Pharm Co., China, CAS: 476-66-4) were freshly prepared in sterile saline solution in a dark bottle.

***Animals***

A total number of 30 adult male Wistar rats (3 months) weighing 100±20 g and ten male young Wistar rats (4 weeks) weighing 25±5 g were obtained from the animal house of the Egyptian Company for Vaccines and Sera (Giza, Egypt). The adult rats were left for seven months without any treatment until the age of 10 months (aged rats) at 22±2 ^°^C and 40-60% humidity with natural light and dark cycles. Within this period, the newborn rats were brought and left for one month until they were two months old at the same temperature and humidity conditions. Five rats were housed in steel mesh cages with water and a commercial pellet diet *ad libitum*. The experiment was performed in the animal house at the Department of Zoology, Faculty of Science, Ain Shams University. All animal experiments were conducted in accordance with the U.K. Animals (Scientific Procedures) Act, 1986 and associated guidelines, EU Directive 2010/63/EU for animal experiments. The study was approved by the Research Ethics Committee for Animal Research at the National Hepatology and Tropical Medicine Research Institute (NHTMRI), research protocol No 18-2018. 

***Study design***

The rats were randomly divided into four groups as follows: Group I (normal adult Control); 10 young rats (2 months old) were orally treated with saline. Group II (Aged Control); 10 aged rats (10 months old) were administered saline orally. Group III (Aged+Ta-65); 10 aged rats (10 months old) were orally treated with Ta-65 supplement (500 mg/kg, (16)). Group IV (Aged+pomegranate); 10 aged rats (10 months) were orally treated with pomegranate extract at a dose of 250 mg/kg (16). All treatments continued on a daily basis for two consecutive months.

***Blood and liver sampling***

At the end of the experimental period, the animals were fasted for 8 hr, weighed, anesthetized with isoflurane, and the blood was immediately collected *via* heart puncture. For separation of serum samples, blood samples were incubated for 20 min at 37 ^°^C, centrifuged at 1500× g at 4 ^°^C for 15 min. Serum was separated, aliquoted, and stored at -80 ^°^C until analysis. A small part of the liver was dispensed in TRIzol for qRT-PCR. The liver was then perfused with sterile isotonic saline through the hepatic portal vein. The liver was then excised, blotted dry with a filter paper, and weighed. The relative weight ratio of the liver in all studied groups was calculated. Small parts from the right lobe of the liver were placed in formalin for light microscopy, and in glutaraldehyde for transmitted electron microscope examination. The remaining liver tissue was placed in a plastic vial containing ice-cold sterile saline and stored at -80 ^°^C until analysis. 

***Biochemical analysis in serum***

The serum levels of total protein and albumin, as well as alanine aminotransferase (ALT) activity, were estimated using commercial colorimetric assay kits (Spectrum, Egypt). Serum globulins level and albumin/globulin (A/G) ratio were then calculated. The IGF-1 level was assayed in the serum using a sandwich ELISA research kit (R&D Systems, USA). The serum level of protein carbonyl was measured as described elsewhere (17).

***Preparation of liver homogenate***

Ten percent of liver tissue was homogenized in ice-cold phosphate-buffered saline (PBS) pH 7.4, using an electric homogenizer (Universal Laboratory Aid MPW-309, Poland). The homogenates were centrifuged at 18,000× g for 20 min at 4 ^°^C (Cooling Microfuge Laborzentrifugen, Sigma, Germany) to obtain the cytosolic supernatant. The cytosolic supernatants were then collected, aliquoted, and stored at -80 ^°^C until analysis. The protein concentration of the homogenates was determined by the method described elsewhere (18).

***Biochemical analysis in liver***

The content of malondialdehyde (MDA) was quantified as described before (19). The activity of total glutathione S-transferase was determined as described before (20). The hepatic active cleaved Caspase-3 activity was determined according to the method of Kamada *et al*. (21) using a colorimetric assay kit obtained from Abcam (UK). 

***RNA extraction from liver tissues, cDNA synthesis, and qPCR***

The total RNA was extracted from the liver tissue using RNeasy Mini Kit according to the instructions of the manufacturer (Qiagen, Germany). The purity of the extracted RNA was assessed at 260/280 nm using UV-spectrophotometer (PhotoBiometer, Eppendorf, Germany). A total of 1 μg of RNA was reverse transcribed into single-stranded complementary DNA (cDNA) using QuantiTect reverse transcription kit (Qiagen, Germany). cDNA synthesis was performed using Gene Amp PCR System 9700 Applied Biosystems (Life Technologies, USA). Quantitative real-time PCR (qPCR) was performed using SYBR green master mix (Qiagen, Germany) to determine the relative expression of thioredoxin reductase 1 (NM_001351984.1), telomerase reverse transcriptase (NM_053423.1), and cytochrome 3a1 ((XM_003751127.4) genes in the liver. Glyceraldehyde 3-phosphate dehydrogenase (*gapdh; *NM_017008.4) was used as a housekeeping gene for normalizing the mRNA expression. qPCR was performed in an optical 96-well plate in a real-time polymerase chain reaction (Agilent Stratagene MX3000P, USA) and cycling conditions (10 min at 95 °C, followed by 50 cycles at 95 ^°^C for 15 sec, 60 °C for 60 sec, and 72 °C for 15 sec). Gene expression was expressed in relative units (RQ = 2^-ΔΔCT^). 

***Localization of p53 in the liver by immunostaining***

Localization and expression of the p53 protein in liver tissue were performed as described elsewhere (22) using mouse anti-rat p53 monoclonal antibody (MA5-12453, Thermo Fischer, USA, diluted 1:20) and goat anti-mouse IgG secondary antibody conjugated with horseradish peroxidase (DAKO Japan Co, Tokyo, Japan, diluted 1:500). Expression of the p53 protein in the liver sections was detected by the reaction of peroxidase with 3,3,9-diaminobenzidine tetrahydrochloride (Sigma, USA), counterstained with hematoxylin. The expression of p53 protein was analyzed using an Olympus microscope and images were captured by a digital Cannon 620 camera.

***Histopathological examination***

The livers from different groups were fixed in 10% formalin solution for at least three days at 4 ^°^C for subsequent staining with hematoxylin and eosin (Sigma, USA). Examination of the slides was performed under a light microscope (Olympus, Japan).

***Transmitted electron microscopy***

Fresh small pieces of liver tissues were fixed in 3% glutaraldehyde and then washed twice in cold phosphate buffer (pH 7.3) for one hour each. The specimens were then post-fixed for two hours in buffered 1% osmium tetraoxide followed by dehydration and embedding in epoxy resin. For selecting optimal areas, semi-thin sections were stained with 25% toluidine blue (Sigma, USA). Ultrathin sections of 60-90 nm were stained with uranyl acetate and lead citrate (23) and examined with an SEO TEM 100 (Sumy Electron Optics Transmission Electron Microscope 100, Ukraine) at a magnification power covering the range from ×5,000 to ×10,000.

***Statistical analysis***

The statistical analysis was performed using the Statistical Package for Social Science version 20 for Windows (SPSS software package, Chicago, USA). The distribution of data was tested using the Kolmogorov-Smirnov test. Data are presented as mean ± SD and their 95% confidence intervals were obtained by nonlinear regression. To compare the difference between the groups, *post hoc* testing was performed by Tukey’s test for multiple comparisons between the different treated groups and their respective controls. *P*-values were considered significant at *P*<0.05. The percentage of change was calculated in comparison with the normal control.

## Results

Aging reduced the body weight as well as the relative liver weight. The treatment of the aged rats with Ta-65 or pomegranate improved body and liver weights. However, this improvement did not achieve a statistical significance (data not shown). Aging did not cause any significant change in serum albumin level or serum ALT activity. Aging significantly (*P*<0.0001) reduced the serum IGF-1 level (-53.34%), compared with the control rats. Aging significantly (*P*<0.0001) increased the levels of total protein (21.54%) and globulins (49.54%) with a significant (*P*<0.0001) reduction in A/G ratio (-30.7%), compared with the control normal rats. Administration of Ta-65 and pomegranate to aged rats normalized the level of serum IGF-1, total protein, and globulins as well as the A/G ratio. Administration of pomegranate to aged rats improved the serum level of IGF-1, compared with the aged rats. However, the level was still significantly (*P*<0.01) lower than that of the normal control animals (Table 1).

Aging resulted in significant (*P*<0.0001) elevations in the hepatic content of MDA (557%) and serum level of protein carbonyl (248%), as well as a significant (*P*<0.0001) decrease in the hepatic GST activity (-36.16%). In aged rats treated with Ta-65 or pomegranate, the hepatic MDA and protein carbonyl contents, as well as GST activity, returned to the normal control level (Table 1).

**Figure 1:**
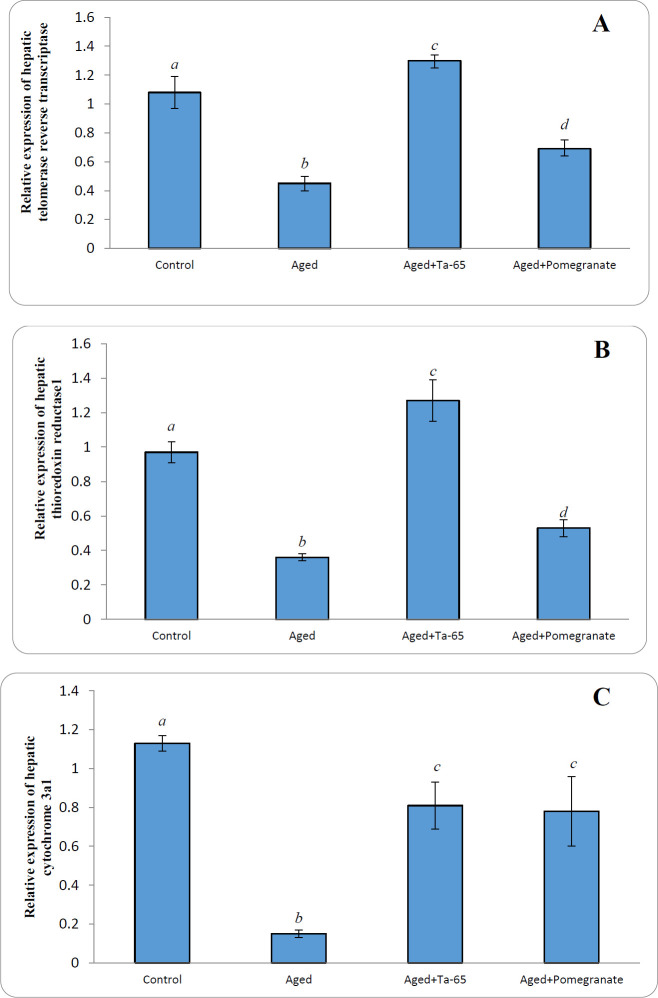
Effect of Ta-65 and pomegranate on relative expression of some genes in the liver of rat

The relative expression of TERT (Figure 1A), thioredoxin reductase1 (Figure 1B), and cytochrome 3a1 (Figure 1C) genes was significantly (*P*<0.0001) down-regulated in aged rats compared with the adult normal rats. Ta-65 administration to aged rats significantly up-regulated the relative expression of hepatic *TERT* and thioredoxin reductase1 (*TrxR1*) genes, compared with both control and aged rats. On the other hand, treatment of aged rats with pomegranate significantly (*P*<0.05) up-regulated the relative expression of *TERT* and *TrxR1*, compared with the aged rats, but the expression level was still significantly (*P*<0.0001) lower than that of the normal adult rats. Administration of Ta-65 or pomegranate to aged rats significantly up-regulated the relative expression of hepatic cytochrome 3a1 compared with aged rats. However, the expression was still significantly lower than that of the control rats.

**Figure 2:**
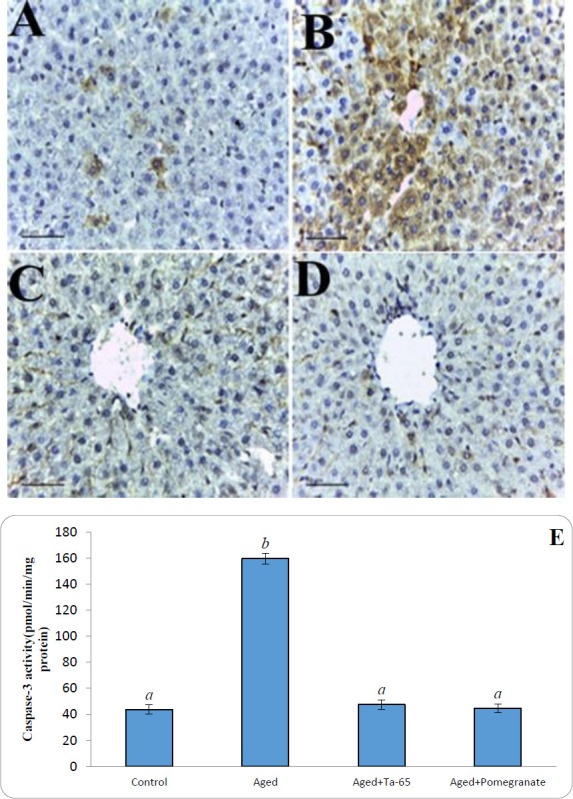
Effect of Ta-65 and pomegranate on the p53 protein (A-D, x200) and Caspase-3 activity (E) in liver tissue of rat

The immunohistochemical analysis of the liver was performed to investigate the localization and expression level of the p53 protein. Results showed weak to negative immunoreactivity (0.075%) of p53 in the hepatocytes of the liver in the control rats (Figure 2A). On the other hand, the immunoreactivity of p53 was increased to severe levels (8.08%) in the liver from the aged rats (Figure 2B). Administration of Ta-65 or pomegranate to aged rats resulted in a significant reduction (0.75 and 0.78%, respectively) in the immunoreactivity for p53 in the hepatocytes (Figures 2C and 2D, respectively). Aging also caused a significant (*P*<0.0001) increase in the activity of hepatic active cleaved Caspase-3 (264.58%). In the aged groups treated with Ta-65 or pomegranate, the Caspase-3 activity returned to the normal level.

**Figure 3:**
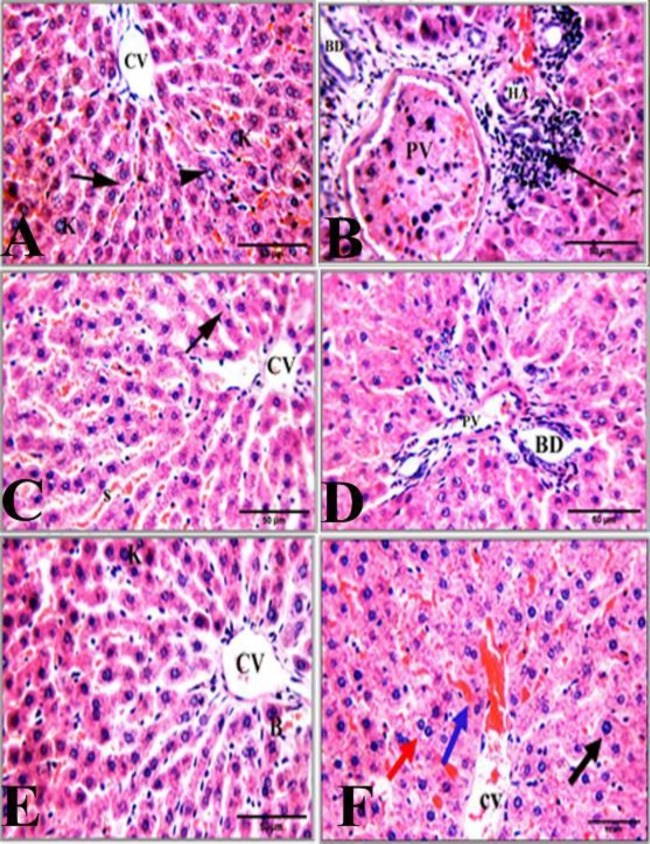
Photomicrographs of rat liver sections stained with hematoxylin and eosin (H&E, 400x)

Liver sections from the adult control rats showed average hepatocytes without any incidence of inflammation or apoptosis (Figure 3A), some hepatocytes were karyomegally and binucleated. The aged rats illustrated the presence of hepatocytes with expanded portal tract with dilated portal vein, inflammatory infiltration, average bile duct, and hepatic artery (Figure 3B). Treatment of aged rats with Ta-65 attenuated the liver histological alterations induced by aging in which normal hepatocytes radiating from the central vein and arranged in single-cell cords were seen, and no signs of apoptosis were observed in the hepatocytes (Figure 3C). The liver central vein, portal vein, portal tract, and bile duct were at the average normal size (Figure 3D). Administration of pomegranate to the aged rats improved the liver architecture; normal hepatocytes radiating from the central vein and arranged in single-cell cords were seen (Figure 3E). No signs of apoptosis were observed in hepatocytes. Furthermore, the portal vein and portal tract were mildly dilated in association with congested blood sinusoids (Figure 3F). The detailed scoring of the liver architecture of all groups is presented in Table 2. It is evident that both Ta-65 and pomegranate were able to mitigate all deteriorations caused by aging. It is also noteworthy that Ta-65 was superior to pomegranate in its alleviative effects.

**Figure 4:**
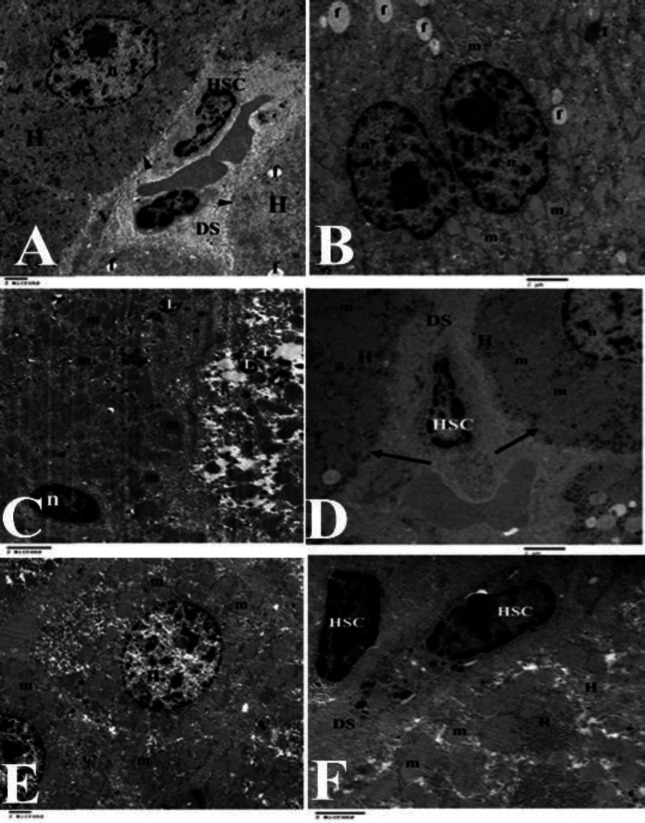
Electron micrographs of the rat liver sections (x5000-10000)

Examination of the liver sections by electron microscope illustrated that the liver of the adult control rats had normal hepatocytes without any signs of apoptosis. Hepatocytes had a euchromatic nucleus, clear numerous mitochondria, unruptured plasma membranes, and rough and smooth endoplasmic reticulum. Normal hepatic stellate cells in the space of Disce were without any sign of collagen strands (Figure 4A). On the other hand, the liver of the aged rats (Figures 4B, 4C) had many apoptotic hepatocytes characterized by the presence of shrunken nuclei. In addition, many hepatocytes had dilated rough endoplasmic reticulum, many lysosomes, and many fat droplets. The aged rats treated with Ta-65 (Figure 4D) or pomegranate (Figures 4E, 4F) showed normal liver ultrastructure. The hepatocytes had a euchromatic nucleus, clear numerous mitochondria, unruptured plasma membranes, normal rough and smooth endoplasmic reticulum, and few lysosomes. No signs of apoptosis were observed in the hepatocytes. The space of Disce had normal hepatic stellate cells without any signs of collagen strands. 

**Table 1:** Effect of Ta-65 and pomegranate on serum liver functions and IGF-1 level as well as markers of oxidative stress in the liver

	Control	Aged	Aged+Ta-65	Aged+Pomegranate
ALT (IU/L)	43.17±4.75^a^	37.83±5.46^a^	40.17±5.56^a^	40.00±7.04^a^
Total Protein (g%)	7.20±0.65^a^	8.75±0.57^b^	7.57±0.27^a^	7.76±0.22^a^
Albumin (g%)	4.27±0.34^a^	4.37±0.23^a^	4.42±0.26^a^	4.21±0.11^a^
Globulins (g%)	2.93±0.40^a^	4.38±0.68^b^	3.14±0.25^a^	3.55±0.30^a^
A/G Ratio	1.47±0.16^a^	1.02±0.19^b^	1.42±0.18^a^	1.20±0.14^a^
IGF-1 (pg/ml)	1802.5±340.1^a^	1037.7±141.9^b^	1894.9±151.2^a^	1327.1±166.5^c^
MDA (nmol/mg protein)	17.22±3.29^a^	113.14±9.43^b^	18.33±2.16^a^	22.35±2.53^a^
GST (µmol/min/mg protein)	5.66±0.39^a^	3.61±0.50^b^	5.86±1.25^a^	5.25±0.52^a^
Protein carbonyl (nmol/ml)	4.8±0.29^a^	16.68±1.31^b^	4.86±0.79^a^	5.19±0.81^a^

Results are expressed as Mean±SD, n= 8. Different characters denote significance (*P<*0.05) between groups

ALT: Alanine aminotransferase, A/G: Albumin/globulin ratio, IGF-1: Insulin-like growth factor-1, MDA: Malondialdehyde, GST: Glutathione S-transferase

(A) telomerase reverse transcriptase, (B) thioredoxin reductase 1, and (C) cytochrome 3a1. Data are presented as Mean±SD, n=6. Different characters denote significance (*P<*0.05)

(A) Control, (B) Aged, (C) Aged+Ta-65, and (D) Aged+pomegranate. Signals appear reddish-brown using DAB as a substrate, (E) Caspase-3 activity. Data are represented as Mean±SD, n=8. Different letters denote significance at *P<*0.05

**Table 2:** Different histological abnormalities in the liver of different groups

Abnormalities	Control	Aged	Aged+Ta-65	Aged+Pomegranate
Dilated CV with Detached Lining	None	++	None	+
Expanded Portal Tract	None	+	None	+
Dilated Portal Vein	None	+	None	+
Karyomegally Hepatocyte	+	++	None	+
Inflammatory Infiltration	None	+++	None	+
Binoculation	+	None	+	+
Steatosis	None	None	None	None
Apoptotic Bodies	None	++	None	None

CV = Central vein; + means mild, ++ means moderate, +++ severe

(A) Control, (B) Aged, (C and D) Aged+Ta65, (E and F) Aged+pomegranate. Central vein (CV) hepatocytes arranged in cords (arrow), karyomegally (K), bi-nucleation (arrowhead), portal vein (PV), bile duct (BD), hepatic artery (HA), apoptotic hepatocytes (a), and blood sinusoids (s)

(A) Control, (B and C) Aged, (D) Aged+Ta-65, (E and F) Aged+pomegranate. Hepatocytes (H), euchromatic nucleus (n), fat droplet (f), Space of Disce (DS), hepatic stellate cell (HSC), microvilli (V), rough endoplasmic reticulum (R), smooth endoplasmic reticulum (s), mitochondria (m), inflammation (I), lysosomes (L), plasma membrane (arrow)

## Discussion

Aging and aging-associated disorders result in the deaths of ~150,000 humans daily (24). The most important of these disorders are neurodegenerative, cardiovascular, hepatic, and renal which severely affect the human quality of life and shorten the lifespan. Although the exact causes of aging are uncertain, accumulations of insults such as oxidative stress, DNA methylation and oxidation, excessive apoptosis, reduced secretion of essential hormones, and disturbed metabolism can result in organ dysfunctions and eventually aging (25). In the current study, we focused on the deteriorations caused by natural aging in the liver and the possible attenuations provided by telomerase activator (TA-65) and pomegranate and their mechanism(s) of action. 

The present study showed that aging did not significantly affect the serum ALT activity or albumin level but resulted in significant elevations in the serum levels of total proteins and globulins. Elevating serum globulins level could be attributed to increasing serum immunoglobulin A (26) due to inflammaging (27). The administration of Ta-65 or pomegranate to aged rats normalized the serum levels of total proteins and globulins due to their anti-inflammatory and anti-oxidant activities (26-29).

The insulin-like growth factor (IGF-1) is produced mainly in the liver to regulate the aging process *via *controlling cell growth and proliferation. A significant decline in the serum IGF-1 level was reported in the current study due to aging in agreement with a previous study (30) and this may be attributed to muscle mass loss (31), lower gene expression (32), oxidative stress, and growth hormone (GH) decline (33)and Forkhead box O1 (FoxO1. GH is the main stimulus for IGF-1 secretion. Treatment of the aged rats with Ta-65 or pomegranate restored the serum IGF-1 level to the normal level. Ta-65 was shown to improve IGF-1 in crossbred sows (34), reduce ER stress, and enhance the IGF-1 pathway (35). Pomegranate increased the capacity of the liver to produce IGF-1 (36).

Oxidative stress and accumulation of free radicals along with disturbance of the anti-oxidant milieu of the cells are some of the main mechanisms involved in development of aging. Many enzymatic and non-enzymatic anti-oxidants are affected by aging. Glutathione S-transferase is a cellular enzyme involved in the detoxification processes of diverse electrophiles. GST is the most accumulated protein in normal cells (37). Thioredoxin reductases (Txnrd1) are a group of selenium-containing oxidoreductases. These enzymes protect the cells from oxidative stress and inflammation (38). Txnrd1 is the only known enzyme that keeps thioredoxin in the reduced form (39). Thioredoxin is pivotal in the formation of the disulfide linkages essential for the activity of many proteins (40). Therefore, thioredoxin and txnrd1 are essential for normal growth and survival of cells. In the current study, aging significantly elevated the hepatic content of MDA and protein carbonyls (markers of lipid and protein peroxidation), reduced GST activity, and down-regulated the expression of* Txnrd1*. These results are in agreement with previous reports (37, 41). Ta-65 and pomegranate restored the normal cellular anti-oxidant milieu and reduced the hepatic MDA and protein carbonyls, elevated the GST activity, and up-regulated the expression of *Txnrd1*. 

The resultant oxidative stress during aging can damage many key enzymes in the metabolic pathways involved in detoxification and drug metabolism. One superfamily of these enzymes is the CYP450 enzymes which are essential for the metabolism and clearance of endogenous metabolites as well as xenobiotics (42). The present study showed that normal aging down-regulated the expression of *cyp3a1* in the liver. Down-regulation of inhibition of CYP450s would lead to accumulation of the drugs, xenobiotics, and environmental contaminants with a severe impact on the liver (43). Inhibition of CYP3A1 (equivalent to CYP3A4 in humans) led to fatal interaction with drugs like astemizole and terfenadine (44)**.** The impact on the older people who are usually on polypharmacy would be more deteriorating. The down-regulation of *cyp3a1* in the liver could be attributed to the significant decline in the liver volume and hepatic blood flow and reduced metabolic rate during aging (45)which might also affect drug metabolism and pharmacokinetics. In addition, the elderly population will develop multiple diseases and, consequently, often has to take several drugs. As the hepatic first-pass effect of highly cleared drugs could be reduced (due to decreases in liver mass and perfusion. Ta-65 or pomegranate improved the expression of hepatic *cyp3a1*. It was reported that Ta-65 significantly increased the *cyp3a1* expression in rats (46). 

The viability of cells and their ability to proliferate and compensate for cell loss is reduced with aging along with an increased rate of apoptosis. Therefore, we investigated the effect of aging on the telomerase reverse transcriptase (TERT) enzyme, p53, and the activity of caspase-3. The TERT enzyme counteracts the shortening of telomeres and therefore plays a pivotal role in aging (47)the end of the chromosomes, and the enzyme telomerase reverse transcriptase (TERT. In the present study, aging reduced the expression of *the TERT *gene. The *Tert* gene expression can be regulated by binding of mitogen-activated protein kinase (MAPK) and extracellular signal-regulated kinases (ERK2) to *TERT *promoters. Aging disrupts these signaling pathways (48)they are not sufficient to drive malignant transformation and require additional events. Frequent co-occurrence of mutations in the promoter for telomerase reverse transcriptase (TERT. Treatment of the aged rats with Ta-65 or pomegranate up-regulated the TERT expression. This up-regulation can maintain telomere length, fight aging, and reverse aging-induced tissue degeneration (49) as opposed to simply arrest, various afflictions of the aged. Such instigators include progressively damaged genomes. Telomerase deficient mice have served as a model system to study the adverse cellular and organismal consequences of wide- spread endogenous DNA damage signaling activation *in vivo*. Telomere loss and uncapping provokes progressive tissue atrophy, stem cell depletion, organ system failure, and impaired tissue injury responses1. Here, we sought to determine whether entrenched multi-system degeneration in adult mice with severe telomere dysfunction can be halted or possibly reversed by reactivation of endogenous telomerase activity. To this end, we engineered a knock-in allele encoding a 4- hydroxytamoxifen (4-OHT. TA-65 can activate telomerase through myelocytomatosis oncogene cellular homolog (c-Myc), MAPK, or ERK pathways *via *binding to the TERT promoter region (50). It was found that Ta-65 increased the telomere length in mice without cancer incidence (38). The tumor suppressor p53 is up-regulated in response to various cellular stressors, including DNA damage, oxidative stress, and aging (51). Up-regulation of p53 induces dramatic and multiple cellular responses, including apoptosis (52). In the present study, aging increased apoptosis in the hepatocytes by up-regulating the expression of p53 protein and elevating the activity of active cleaved caspase-3 indicating that apoptosis is elevated. This was confirmed by the histological studies in which many apoptotic hepatocytes were observed with chromatin condensation and shrunken nuclei. During aging, the generated reactive oxygen species (ROS) can promote apoptosis (53)but the mechanism remains unknown. Similarly, the Bcl-2 oncoprotein can suppress PCD in a variety of cell types and circumstances, but it is not known how it does so. It has been suggested that PCD involves the generation of reactive oxygen species (ROS. Treatment of the aged rats with Ta-65 reduced the expression of p53 protein near to the normal expression level and reduced the activity of caspase-3 in the liver. These findings were confirmed by histological and ultrastructure examinations. The activation of telomerase by Ta-65 was reported to reduce ROS and DNA damage and subsequently reduce p53 in aging-induced ischemic skeletal muscle (54)which is regulated by telomerase. We examined the role of telomerase activity during impaired collateral growth during aging in ischemic skeletal muscle. Unilateral hind limb ischemia was generated in old, young, and old mice chronically administered a telomerase activator. In old mice, blood flow recovery and capillary density development in ischemic hind limbs were reduced compared to those in young mice, and these changes were restored to equal levels by administration of TA-65, a telomerase activator. During the early phase of ischemic muscle changes in old mice, telomerase reverse transcriptase expression and telomerase activity were both low compared to those in young mice and old mice treated with TA-65. Levels of reactive oxygen species (ROS. It was reported that treatment with *A. membranaceus* polysaccharides attenuated the cardiac apoptosis and necrosis induced by diabetes through affecting the expression of peroxisome proliferator-activated receptor γ coactivator 1α or inhibiting the expression of brain natriuretic peptide (29). These remain assumptions and we did not investigate these pathways in the present study. Treatment of the aged rats with pomegranate reduced the hepatic p53 protein in association with a decline in the hepatic Caspase-3 activity. These findings prevented the aging-induced excessive apoptosis manifested by the histological findings. However, the attenuation provided by TA-65 was superior to that provided by the pomegranate. Hassanen *et al*. (55) particularly in the Middle East. It is an essential commercial crop full of bioactive compounds with several medical applications. Pomegranate is very popular for its biological effects exerted by phenolic compounds via free radical scavenging abilities. It has revealed high anti-oxidant and anti-inflammatory activities and is beneficial for the amelioration of liver and kidney diseases. Purpose: To elucidate the potential efficacy of pomegranate juice (PJ reported that pomegranate diminished the mitochondrial-dependent apoptosis *via* increasing Bcl-2 expression, decreasing the caspase-3 expression in the liver. The protective effect of pomegranate was attributed to its phenolic compounds comprising phenolic acids, tannins, flavonoids, and anthocyanins (56).

## Conclusion

Aging is a multifactorial natural process. Aging resulted in inflammation, disturbance of hormones and metabolic enzymes, oxidative stress, loss of cell viability, and enhanced apoptosis. Ta-65 and pomegranate supplements have anti-aging activities against aging-induced liver deteriorations. Ta-65 and pomegranate elevated the expression of *TERT*, *txnrd1*, and *cyp3a1* genes in the liver, increased the IGF-1 level, alleviated the oxidative stress, and reduced apoptosis. This was manifested in the architecture of the liver. Ta-65 was superior to pomegranate in many aspects. Therefore, Ta-65 and pomegranate supplements are promising therapeutic agents for delaying the deteriorations induced by normal aging and merit further investigations. 
